# Editorial: New Insights into Estrogen/Estrogen Receptor Effects in the Cardiac and Skeletal Muscle

**DOI:** 10.3389/fendo.2020.00141

**Published:** 2020-03-19

**Authors:** Dawn A. Lowe, Georgios Kararigas

**Affiliations:** ^1^Department of Rehabilitation Medicine, University of Minnesota, Minneapolis, MN, United States; ^2^Charité – Universitätsmedizin Berlin, Corporate Member of Freie Universität Berlin, Humboldt-Universität zu Berlin, and Berlin Institute of Health, Berlin, Germany; ^3^DZHK (German Centre for Cardiovascular Research), Partner Site Berlin, Berlin, Germany

**Keywords:** cardiovascular disease, estrogen, heart, receptor, skeletal muscle, steroid

Decreased levels of the steroid hormone estrogen at menopause are associated with an increased incidence of cardiovascular disease and loss of skeletal muscle mass and strength. Consequently, it has been generally expected that estrogen may be a crucial protective factor against the development of cardiovascular disease and that it may be implicated in the regulation of skeletal muscle function in women. Estrogen signals through the classical nuclear estrogen receptors (ER) α and β, as well as the membrane G protein-coupled receptor GPR30 (also referred to as GPER), via the genomic or non-genomic pathway.

Studies reporting actions of estrogen in the heart show direct cardiac estrogenic effects (1, 2), which may differ significantly between the sexes (3–6). In this Research Topic, Ueda et al. provide an overview of ER signaling in the cardiovascular system, including cardiac myocytes and fibroblasts. Considerable effort has focused on non-nuclear ER signaling and non-genomic effects of estrogen. Accordingly, Puglisi et al. review non-genomic effects of estrogen on cell homeostasis and remodeling focusing on cardiac ischemia/reperfusion injury.

Mitochondrial bioenergetics are at the core of cardiac and skeletal muscle function. Ventura-Clapier et al. provide an overview of the effects of estrogen and its receptors in cardiac and skeletal muscle mitochondria. Mahmoodzadeh and Dworatzek take a closer look and review the regulation of cardiac mitochondrial function and Ca^2+^ ion channels by 17β-estradiol (E2) and its receptors, thereby affecting contractile function. The E2/ER axis also impacts skeletal muscle contractility and mitochondrial bioenergetics (7–11). Counts et al. report in their original research study that mitochondrial dysfunction was attenuated by the administration of E2 in a genetic mouse model of cachexia. In addition to regulating mitochondrial bioenergetics, estrogen is expected to confer protection against oxidative stress. In their original research article, Ogola et al. provide insight into how acute estrogen signaling via GPER provides cardiovascular protection in angiotensin II-induced hypertension characterized by increased oxidative stress.

The decline of estrogen at menopause is associated with changes in several cardiovascular risk factors, including the atherogenic lipid profile and calcification in cardiovascular structures. In this context, Zhang et al. provide a review of the effects of estrogen in basic biological pathways associated with vascular and cardiac valvular tissue calcification, as well as potential strategies of pharmacological therapy to reduce or slow these processes. Karvinen et al. investigated whether physical activity attenuates changes in the atherogenic lipid profile and cardiovascular risk in postmenopausal women. Their results suggest that physical activity may attenuate menopause-associated atherogenic changes of healthy middle-aged women to a certain extent. Interestingly, physical activity, in turn, is affected by estrogen (12, 13).

The E2/ER axis may also affect immune responses, thereby affecting the risk of infection and subsequent development of inflammatory heart disease, such as myocarditis, which may lead to cardiomyopathy and heart failure. In their original research study, Bruno et al. found that exposure to the endocrine disruptor bisphenol A led to altered ER expression in the heart, suggesting an increased risk of developing myocarditis after a viral infection in females.

Pronounced sex differences exist in the development and pathophysiology of cardiovascular diseases (14), such as pressure overload-induced left ventricular hypertrophy (15–20), as well as the response to therapy (21, 22). Estrogen is thought to play a major role in cardiovascular sex differences. Notably, it has been put forward that the decrease of estrogen at menopause may be a contributor to the development of heart failure with preserved ejection fraction (23), which mostly affects women (24). Considering the potential underlying pathomechanisms, Sickinghe et al. propose the hypothesis that the menopause-related estrogen decline contributes to myocardial microvascular dysfunction and they provide an overview of molecular targets of estrogen that might guide future research and treatment options. Along this line, Groban et al. outline the impact of GPER on diastolic function, left ventricular stiffness and aortic distensibility, among others.

Collectively, this Research Topic includes clinical- and pre-clinical studies in original research and state-of-the-art review articles focusing on the effects of estrogen on cardiovascular physiology and skeletal muscle biology and function. Detailed characterization of the regulation of (patho)physiology by estrogen and its receptors in cardiac and skeletal muscle, along with elucidation of the underlying mechanisms may lead to the identification of novel therapeutic targets, which may have a wide implication in the development of new and personalized therapies.

## Author Contributions

GK conceived the work and drafted the manuscript. DL revised critically the manuscript. DL and GK read and approved of the submitted manuscript.

### Conflict of Interest

The authors declare that the research was conducted in the absence of any commercial or financial relationships that could be construed as a potential conflict of interest.
